# The Hospitals Association

**Published:** 1888-06-30

**Authors:** Prebendary Barnes


### The Hospitals Association.

Speeches by Dr. Steele (of Guy's), Mr. Nixon (of the London), and the Rev. Prebendary Barnes.

A general meeting of the members of the Hospitals Association was held on Wednesday, June 20th, 1888, at St. Thomas's Hospital, in order to resume the discussion upon the paper read by Mr. Burdett-Coutts, M.P., at the preceding meeting, upon "The Pay System in Hospitals." Dr. Bristowe, F.R.S. (the President), presided.

Dr. Steele, the Medical Superintendent of Guy's Hospital, rose to resume the debate. He said: Since our last meeting in this hall the subject of patients' contributions to hospitals has been pretty freely discussed out of doors by the public press; but I doubt whether the discussion has elicited much more light than we had elicited at our meeting by the aid of Mr. Burdett-Coutts's excellent and exhaustive paper. On that occasion, apart from Mr. Burdett-Coutts's paper, we had speeches from some very eminent authorities in hospital administration, and I fancy that they rather objected or were indisposed to fall in with the idea of self help as applied to the old-fashioned hospitals, although they had no objection to seeing the principle introduced into new hospitals. (Hear, hear.) I felt that these gentlemen had not had the practical experience of the pay system which I myself had had, and during the last few years I have been very much exercised in my own mind in regard to the self-help system. The hospital I represent introduced this system four or five years ago. It has, I think I may say, been in existence for about five years, and has worked remarkably well. It consists in charging our out-patients a few pence for their weekly supply of medicine, and in making a higher charge to persons in a higher walk of life, such as clergymen, medical men, &c., who could afford to pay a guinea a week. The system is not entirely limited to Guy's Hospital, for it has extended about the country as well as in London, and has chiefly been adopted in London in the special hospitals. No other general hospital, except Guy's, has taken this question up in the sense in which I have spoken. The statistics which are published by the council of the Hospital Sunday Fund show that during the last year no less an amount than £42,000 was collected from patients in hospitals. That is a large sum, and if the system had fair play I have not the slightest doubt the amount could be multiplied four or five times. Then, we can scarcely exaggerate the great importance of the moral argument of this self-help system. I cannot help coming to the conclusion arrived at by great political economists, as well as by very many good men and women who look a little beneath the surface, that indiscriminate medical relief has very much the same effect upon the population as indiscriminate almsgiving, and demoralises the population and robs the labour market. In no other place will you find such a number of free medical charities as there are in London, and in no other place will you find such a system of medical organisation. (Hear, hear.) It may be said, Should not persons who participate in the benefits of hospitals be required to pay something towards their maintenance? Many opportunities are given to patients in hospitals to contribute by means of contribution boxes, but the result of that is practically *nil*. We placed three contribution boxes in our out-patients' department, through which 60,000 patients pass every year, and the duty of opening those boxes upon New Year's Day devolved upon me. The proceeds of those boxes—the contributions of 60,000 people—amounted to an average of 30*s*. One year the amount was £2 10*s*., but I explained that by the fact that some one had dropped a sovereign in by mistake. If I am wrong I beg the donor's pardon. I would charge these people a few coppers for their medicine, but I should be very sorry to adopt any system of coercion—very sorry indeed. I should allow the people who said they were unable to pay to get their medicine for nothing in the first instance, but when they came again the week after, as they generally do, I should require them to produce some testimony as to their inability to pay from a respectable householder, a clergyman, or from the Charity Organisation Society. As I said before, indiscriminate or promiscuous medical relief not only demoralises the recipient, but also robs the labour market. People do not value much what they get for nothing, and probably no provident dispensary thrives much in the neighbourhood of hospitals. People who frequent hospitals are composed of various classes of the community, and very few indeed can be said to belong to the destitute classes. About three-fourths belong to a class who can afford a little—who pay twopence for their glass of beer and find the money for their railway fare. I have seen them at the refreshment buffet which we have at the hospital indulging in sandwiches, and coffee, and sausage rolls. Why should they not also pay something for their medicine—their "bottles of stuff," as they call it, and to get which they have frequently travelled a considerable distance, and lost half-a-day's work in the bargain? I am afraid the managers of our public institutions are making a mistake in not adopting some new system in regard to the admission of patients. Our present system requires improvement very much indeed. It has been very good in its day; but while other systems go on improving in the present day, this system remains in the same condition year after year. Self-help should be introduced into its constitution, and it is the duty of those in authority to stimulate and encourage this habit of self-denial and independence which, if properly worked upon, would relieve one-half of the misery of the world. (Cheers.) I cannot sit down without personally thanking Mr. Burdett-Coutts for his able and exhaustive paper. (Cheers.) I am sure it will greatly aid the cause of charity in the highest sense of the word—charity judiciously administered. (Cheers.)

Mr. W. J. Nixon, House Governor of the London Hospital, said: I was not prepared to speak in this discussion to-night, but since the last meeting I have been reading this paper very carefully, and have particularly noticed the remarks with which the lecturer summed up the question, and gave his excellent opinion on certain matters which he supports by arguments. I wish to say a few words on the other side of the question. I would say, in the first place, that I consider Mr. Burdett-Coutts has conferred a great favour upon all hospitals, and upon all who deal with the administration of those institutions, by his paper. I do not wish it to be supposed that I am in opposition to the principle of self-help, but I wish to point out to you the degree beyond which you cannot expect self-help to go. I am most anxious that we should be relieved from our difficulties. It is quite impossible, in the ten minutes allotted to each speaker, to say one-tenth part of what one would desire upon the multitude of subjects which Mr. Coutts has raised. As far as the principle of self-help and the Pay System can be introduced, by all means try the experiment; but all the large general hospitals, which are founded on the principle of charity, should be left untouched, and should be kept on the basis they now occupy. (Hear, hear.) I cannot see how the system could aid the London Hospital. I have been connected with the London Hospital for over forty years, and I never knew the time when that hospital, or any other public hospital, was not in debts—that is to say a hospital which is not supported by endowments. I believe I am the originator of the advertisement "Funds urgently needed." I remember drawing the advertisement up, and as there was no patent for it, it has gone on all over the world ever since. It is stated that legacies are an unreliable source of income. I think to the contrary. They are extremely fluctuating, but they are extremely certain in the long run, and all well-managed charities may look forward to a large amount of legacies for the simple reason that those who cannot afford to give while they are alive look round and pick out good charities to which they can leave the money after death which they would have liked to give before. In fact, while we always have the poor with us, we also always have the rich with us. I think the incidence of charity is wrong where it falls upon improper subjects, and we should all try to abolish those parts of the system which are wrong in principle and favour improper persons in the application. We are trying to do that at the London hospital. I think multitudes of persons go to free hospitals who have money in their pockets. They could afford to pay good round sums at a special hospital, but could not afford to pay the large fees of a special practitioner.

Mr. Burdett-Coutts, M.P.: The gentleman now speaking has said that the London Hospital has endeavoured to get rid of any bad part of the free system. By his preceding remarks I understand him to mean that the London Hospital has endeavoured to avoid giving its assistance to all those who are not absolutely poor. The whole question which I wish to put is this: What means do they adopt to find out that their patients are absolutely poor?

Mr. Nixon: About five years ago I obtained permission to commence a system of inspection which should prevail over the whole of the out-patients of the hospital. You cannot inspect the in-patients in an institution where over three-fifths of the people are taken in on the spot. In the out-patients' department, in which from 60,000 to 80,000 patients come under our treatment annually, I have established a system which has worked efficiently. In our room stands an inspector, who knows every doubtful case. If his doubts are confirmed he says, "I shall make inquiries into your circumstances. You must give a reference. You may receive your first treatment; but when you come again you must bring proofs as to your needy position." If it is a case of genuine neccessity, the people come again, and receive treatment. If it is not, they are ashamed, and do not come. I agree entirely that the pay system should mean that every person should pay what he can; but it is impossible to carry out that system where you give relief upon no other principle except that of real charity. Mr. Burdett says governors' letters are not the pay system. No, but they form a system established by persons who decided to invest their money in a particular way, the interest of which should go to those who were too poor to procure a bed for themselves in a hospital. You cannot clear the wards of people who are relatively well-to-do; but we tried, and I think have succeeded in it, to keep all people out from the out-patients department who were able to pay. Our eight hundred beds are generally overflowing with poor patients, and we cannot put a ward on one side for people who can pay. I have no doubt the general system of paying patients could be carried out if it were put upon the American plan, but you must first show us where and how it can be applied. There must always be a certain number of beds kept vacant of different classes of patients as they come in, and it is impossible to further subdivide the wards. I think that payment for out-patients damages everybody concerned. The poor people, if they pay for advice, are supposed to be fogging the doctors, and the general run of hospital practitioners object very highly to be considered as receiving payment for the attendance of poor people, and glory in giving their work in the most perfectly gratuitous manner. If you give them a prescription, and they go to a chemist and get it made up, they generally get very inferior materials. We had one instance in which a man who had an eye complaint had been given a prescription to get made up as a liquid in which to bathe his eyes, and we actually found that he was bathing them in a solution of Epsom salts. Then, if we were to charge for the material, the charges would very often be more than a poor man ought to be required to give. The average payment for drugs, &c., may be estimated at 1½*d.* per day per patient, including the in-patients. But sometimes the patients would have to pay considerably more for what they got; and although I do not wish to shock you with bad language, I can myself imagine the many sanguinary words which would be used by a coalheaver who would be called upon to pay what he would term "half a bob," for what he could get at the chemist's for ½*d.*

A Gentleman in the Meeting: What wages would the coalheaver get?

Mr. Nixon: That is a question which I cannot now go into. In Mr. Burdett's paper it is suggested that we should carry out a system by which general paupers should be ad mitted who cannot be treated in poor-law infirmaries. That system is truly carried out. We always charge the unions with cases which belong to them and which we treat in the wards. When we can get the money we always do. I think I may sum up by saying that all general hospitals are, as a matter of fact, charities, and while they have their charters they are bound to proceed upon that principle. If the pay system would improve them, I should like to see it effectively and properly carried out.

The Rev. Prebendary Barnes: I should like to take up the words of the last speaker. Hospitals are great institutions, no doubt, but hospitals, as institutions of Great Britain, are not necessarily direct charities, and there has undoubtedly come a time when we must look to the question of dealing with the hospitals very seriously. The deficit, if it were paid off at once, would occur again. The difficulty is a great one, and it belongs to us at the present time to consider it, the paying system being the only one means of several others by which we are likely to be able to meet it. I would refer you to what was the case twenty years ago, when the late Lord Iddesleigh, then Sir Stafford Northcote, was thinking of benefit societies. He found the subject a most difficult one, and it was hardly possible to see how the thing could be arranged, even by legislative action. His lordship said, "Let there be no legislation, but let there be an inquiry which Government assists and which has the power of calling witnesses." The result was legislation which has been universally regarded as a great piece of statesmanship. It would be quite possible for Government, with great expenditure of public funds, to classify and arrange hospitals so that when they come to be dealt with by the Legislature the whole of the facts with regard to them should be thoroughly known and made public. It is an essential part of the question whether statesmen like Mr. Burdett-Coutts are required to understand that this matter is part of the government of England; and when we have got as far as this, we shall see that if this matter were looked at in this way it might be quite possible to have a Royal Commission which should have the power to call witnesses, which should tabulate the hospitals and infirmaries and dispensaries throughout the kingdom, and which should state to the public in the fullest possible terms what steps should be taken with each. We find that the largest amount of sick poor are in State-aided hospitals, infirmaries, and sick-wards attached to our unions, and all these are State-aided, and therefore necessarily State-controlled, because State-control is a consequence of State-aid. Then we get an unhappily increasing class of patients suffering from diseases of the mind which are treated under the control of, and with the benefit of, State-aid. I do not think we could have had a stronger speech in support of the system which Mr. Burdett-Coutts has laid before you than that which we have just listened to from Mr. Nixon—a gentleman with a really thorough and exact knowledge of the subject of hospital administration. I conclude by saying what I commenced with, that this is a statesmen's question, and we should do well to support in every possible way the Hospitals Association, which has undertaken the very difficult task of putting this before the public, so that whenever the Government does take up the matter, the amount of facts which the Association has obtained will be most useful for the purposes of a general inquiry.

*(To be continued.)*

